# Socioeconomic factors associated with tobacco smoking among adult males in Sri Lanka

**DOI:** 10.1186/s12889-019-7147-9

**Published:** 2019-06-18

**Authors:** Hiranya Nilakshi Fernando, Imaesha Thilini Pradeepika Wimaladasa, Anjali Nimaya Sathkoralage, Ashani Nisansala Ariyadasa, Chathurika Udeni, Lahiru Sandaruwan Galgamuwa, Prasanna Herath, Nishantha Kumarasinghe

**Affiliations:** 1 Department of Nursing and Midwifery, Faculty of Allied Health Sciences, General Sir John Kotelawala Defence University, Colombo, Sri Lanka; 2 Department of Medical Laboratory Sciences, Faculty of Health Sciences, The Open University of Sri Lanka, Colombo, Sri Lanka; 3 Department of Pre-Clinical Sciences, Faculty of Medicine, General Sir John Kotelawala Defence University, Colombo, Sri Lanka

**Keywords:** Tobacco, Taxation, Smoking

## Abstract

**Background:**

Tobacco smoking is considered as a major public health issue worldwide. Reduction of tobacco usage has been one of the main government policies in Sri Lanka and the price of cigarettes has been raised several times in the last few years. The purpose of this study was to evaluate the socioeconomic factors associated with tobacco smoking among adult males in Sri Lanka.

**Methods:**

A study was conducted in Gampaha district in Sri Lanka recruiting 365 tobacco smoking people and their spouses. Data regarding tobacco smoking were obtained using an interviewer administrated questionnaire.

**Results:**

Frequency of tobacco smoking was negatively associated with the improvement of educational levels. Employment, monthly income, influence of friends, smoking frequency before price increment, weekly expenditure for smoking, low educational level and the age of first smoking exposure was significantly associated with tobacco smoking among smokers. According to the spouses, smoking frequency before price increment, weekly expenditure of the husbands of smoking and influence of friends, number on smoking friends, spouse’s employment and husband’s monthly income were factors associated with tobacco smoking of their husbands. In addition, smoking at home, at work places and at friend’s houses was significant with the frequency of daily smoking.

**Conclusions:**

Increasing the price of tobacco products has no significant impact on smoking behaviors in Sri Lanka. The need for essential strategies to educate and motivate the smokers to stop smoking is required. Primary care health workers might play a major role in motivating smokers to quit smoking.

## Background

Smoking of tobacco products occurs occasionally or habitually as a consequence of a physical addiction to some chemicals, primarily the highly addictive psychoactive ingredients such as nicotine [1]. More than 1 billion people are smoking globally and 80% of them are living in low and middle income countries [2, 3]. In Sri Lanka, it is recorded as 29.9% individuals are currently smoking [4].

Tobacco is the second major cause of adult mortality in the world. The number is expected to exceed 8 million deaths by 2030, with approximately 70% of these deaths are occurring in developing countries [5]. Smokers also face a much greater risk of premature death than non-smokers [6]. In addition, smoking tends to cause accelerated age related cognitive decline associated with the loss of grey matter in the brain [7].

Tobacco smoking increases the risk of developing chronic obstructive pulmonary diseases (COPD), particularly in high and middle income countries [4]. Frequency of smoking is increasing as economies develop, but it is also linked to poverty and poor education [4]. Breathing unhealthy air is a risk factor for most respiratory diseases [8]. The key factors for preventing such diseases are reduction of tobacco smoking and the improvement of air quality, which includes a reduction in second-hand or passive tobacco smoke inhalation.

The government of Sri Lanka has introduced many anti-smoking programs to reduce the consumption of tobacco products among Sri Lankans. Prohibition of sales and promotion of tobacco products to minors (below 21 years), prohibition of advertising, promotion and sponsorship, compulsory health warnings on cigarette packets, theatres and television programs and prohibition of smoking in public places are the important provisions to reduce the usage of tobacco products in Sri Lanka. In addition, the policy of price increase aiming to discourage the consumption of cigarettes was introduced to the country after 2010.

The marital relationship directly influences to the health behaviors of the wife and husband [9]. Many studies have documented that married people have better health and healthy behaviors than the unmarried [10, 11]. However, sparse information is available to assess the awareness of socioeconomic factors of tobacco smoking in Sri Lanka. Therefore, this study was designed to evaluate the association of socioeconomic factors with tobacco smoking among adult males and the awareness of spouses about smoking of their husbands in Sri Lanka.

## Methods

### Study setting/population

This study was conducted in Gampaha district from June to December 2017. Gampaha District spreads the area of 1387 km^2^ and is located in the western province of Sri Lanka adjacent to the commercial capital, Colombo. The population of Gampaha district was nearly 2.3 million in 2017. This district has been divided into 15 Medical offices of Health (MOH) divisions. Out of them, 10 divisions were selected randomly using SPSS statistical software for this research namely; Gampaha, Mirigama, Divulapitiya, Aththanagalla, Negombo, Kelaniya, Ragama, Minuwangoda, Biyagama and Ja-Ela.

The sample population was randomly selected from 10 MOH areas in Gampaha district. The data were collected with the support of regional health officers such as Public Health Nursing Sisters (PHNS), Public Health Midwives (PHM), Public Health Inspectors (PHI) and Grama Niladari (GN) officers in the selected areas. They assisted to coordinate this study to recruit married couples and was explained the objectives and the procedure of the study.

### Sampling and sample size

The sample size was calculated using the equation of *n* = z^2^ p (1-p) / d^2^ where *n* = sample size, z = 1.96; critical value of specified confidence at 95% confidence interval, *p* = probable estimate of proportion of the prevalence of tobacco smoking among males in Sri Lanka (29.9%) [4] and d = 5% of absolute error. Minimal sample size was calculated as 330. In addition, 10% sampling error was added to minimize irresponsible and recording errors and the final sample size was 365.

Then, 365 married couples were selected from the selected MOH divisions. The number of couples in each division was considered according to voter registration of each MOH division. Then written consent forms were obtained from selected smoking adult males to participate in the study. Males, who were aged below 18 years and unmarried, were excluded. In addition, spouses of the selected smoking males were included for the study.

## Data collection

Information on tobacco consumption was obtained using an interviewer administered questionnaire. Before the initiation of each interview, the interviewer explained the objectives of the study. After obtaining informed written consents, data was collected from male smokers and their spouses at their residences. The questionnaire consisted of a mixture of qualitative and quantitative questions. Male questionnaire consisted with 33 questions regarding socio demographic data, smoking history and smoking behavior; economic factors contributed to tobacco smoking and preferred places of tobacco smoking. Female questionnaire consisted with 35 questions regarding socio demographic data, awareness of duration, influence and types of husband’s smoking, economic factors contributed to husband’s tobacco smoking, spouses attitudes regarding tobacco smoking, spouses awareness of husband’s preferred smoking places. The questionnaire was initially developed in English and then translated into the native languages in Sri Lankans (Sinhala and Tamil).

### Statistical analysis

Ages of the participants were categorized into three groups; 19–39, 40–59, 60–79 years old. Educational attainment was classified into three groups; who were completed ordinary level or below ordinary level (11 years or low school education), completed advanced level (13 years of school education) and completed Diploma or Degree level (more than 13 years of school education).

All data was entered into a Microsoft Excel 2010 data sheet. Data was analyzed by descriptive statistical analyzing methods using SPSS Version 23. Male smokers were divided into two groups as daily smokers and not daily smokers depending on their frequency of smoking. Multivariate logistic regression analysis was applied to assess the association of socioeconomic factors with tobacco smoking. Chi square test was used to determine the preferred places for smoking among smokers. *P* value less than 0.05 was considered as significant.

## Results

### General characteristics

Three hundred and sixty five married male smokers (mean age 43.3 ± 11.5 years) and their spouses (mean age 40.2 ± 11.1 years) were participated to the study. Majority of the participants educated up to the general certificate of education (GCE) ordinary level. Majority of the males were employed (98.9%) while 69% females were unemployed. Most of males (44.9%) and females (54.9%) were employed less than 10 years. Mean monthly income for males was 48,170 ± 60,053 LKR (Sri Lankan Rupees) (267.6 ± 333.6 USD) and for females was 34,796.50 ± 32,351 LKR (193.3 ± 179.7 USD).

### Smoking patterns and behaviors of smokers and awareness of their spouses

Mean age of the first experience of smoking was 16.7 years. Interestingly, two thirds of smokers had more than 10 smoking friends. Majority of smokers (83.2%) used premade cigarettes and “Gold leaf” was the commonest brand of cigarette among these participants. Majority of the male participants (69.9%) were daily smokers before increasing the price in 2016 and majority of the study participants (93.4%) were aware of the new price. However, 69.3% participants smoked daily after increasing the price. In addition, 59.2% of participants reported that they smoked similar frequency of tobacco products per day before and after price increasing. More than 80 % of smokers were not used any alternatives instead of tobacco products after raising the price of tobacco products.

More than two third of spouses were aware of smoking of their husbands before marriage. According to 92.1% of spouses, the influence of friends is very important factor to tend them for smoking. Cigarette is the most common tobacco product and Gold leaf is the commonest brand of cigarettes in Sri Lanka.

More than half of male participants prefer to smoke at home, at friend’s house, at social event and other places like parties. However, in 59% of spouse’s opinion, home is the preferred place for smoking for their husbands. Majority of both male (84.9%) and female (84.4%) participants expressed that they don’t smoke at public places.

In accordance with the awareness of spouses, more than half of (56.7%) their husbands were smoking daily before price increment. Out of them, 66.3% continued to smoke daily after increasing the price of tobacco products. Majority of the spouses (86.6%) reported that their husbands smoke less than 10 cigarettes per day after the price increment. More than half of female participants were not happy about the proportion of income spends for household activities. According to half of spouses, smoking is the main reason for a low proportion of income available for house hold activities.

### Associations of socioeconomic factors with tobacco smoking among adult males

Multivariate logistic regression analysis identified that frequency of smoking before and after price increment, weekly expenditure for smoking, employment of the smoker, monthly income, influence of friends, low educational level and the age of first smoking exposure was significantly associated with tobacco smoking (Table 1). In addition, smoking at home, at work places and at friend’s houses were also significant factors with current rate of smoking per day (Table 2).

**Table 1:** Association of socioeconomic factors with tobacco smoking (Male)

Variable	Categories	Current Frequency		OR	95% CI	*p* value
		Daily (%)	Not daily (%)			
Age (Years)	19–39	99 (38.8)	52 (47.3)	1		
	40–59	125 (49.0)	44 (40.0)	0.462	0.125–1.711	0.248
	60–79	31 (12.2)	14 (12.7)	0.355	0.108–1.160	0.086
Educational level	Up to Ordinary level	159 (62.3)	52 (47.3)	1		
	Up to Advanced level	66 (25.9)	35 (31.8)	0.377	0.117–1.216	0.103
	Diploma/Degree	30 (11.8)	23 (20.9)	0.140	0.036–0.427	0.041
Employment	Government	119 (45.8)	55 (50.0)	1		
	Private	70 (26.1)	33 (30.0)	2.431	0.862–6.855	0.093
	Self-employment	66 (26.1)	22 (20.0)	5.036	1.632–15.545	0.005
Duration of employment (Years)	< 1	17 (6.7)	5 (4.5)	1		
	1–10	107 (41.9)	56 (50.9)	0.204	0.025–1.637	0.134
	11–20	82 (32.2)	32 (29.1)	1.263	0.404–3.949	0.688
	> 20	49 (19.2)	17 (15.5)	1.164	0.364–3.719	0.798
Monthly income (LKR)	< 10,000 (< 55.5 USD)	12 (4.7)	2 (1.8)	1	0.462-1.855	
	10,001–50,000 (55.5–277.7 USD)	174 (68.2)	68 (61.8)	0.302		0.419
	50,001–100,000 (277.7–555.5USD)	61 (23.9)	34 (30.9)	0.081	0.012–0.568	0.011
	> 100,000 (> 555.5 USD)	8 (3.1)	6 (5.5)	0.106	0.016–0.724	0.022
First exposure of smoking (Years old)	< 18	134 (52.5)	44 (40.0)	1		
	> 18	121 (47.5)	66 (60.0)	0.418	0.188–0.933	0.033
Influence to smoking	Self-preferred	62 (24.3)	24 (30.8)	1		
	Friends	163 (63.9)	62 (28.0)	0.120	0.030–0.484	0.003
	Others	30 (11.8)	23 (43.2)	0.219	0.071–0.670	0.008
Current form of tobacco	Cigarette	172 (67.5)	86 (78.1)	1		
	Cigar	6 (2.3)	1 (0.9)	1.470	0.577–3.741	0.419
	Beedi	17 (6.7)	3 (2.7)	0.254	0.010–6.742	0.413
	Others	60 (23.5)	20 (18.2)	0.362	0.057–2.311	0.283
Form of cigarette	Premade cigarette	140 (81.2)	73 (84.5)	1		
	Roll on your own	23 (13.3)	10 (9.1)	0.506	0.104–2.465	0.399
	Both	9 (5.5)	3 (5.4)	0.552	0.206–1.435	0.106
Types of cigarette	Gold leaf	80 (46.6)	36 (42.0)	1		
	Bristol	30 (17.6)	13 (15.2)	1.018	0.429–2.419	0.970
	Others	62 (35.7)	37 (40.2)	0.420	0.139–1.272	0.125
Past frequency	Daily	223 (87.4)	33 (30.0)	1		
	Not daily	23 (9.0)	72 (65.5)	0.273	0.049–1.509	0.137
	Never	9 (3.5)	5 (4.5)	10.396	1.779–60.868	0.009
Weekly expenditure (LKR)	< 250 (< 1.4 USD)	54 (21.2)	60 (23.5)	1		
	250–500 (1.4–2.8 USD)	92 (36.0)	23 (20.9)	16.705	4.446–6.765	< 0.001
	501–1000 (2.8–5.6 USD)	46 (18.0)	21 (19.1)	1.993	0.555–7.155	0.290
	> 1000 (> 5.6 USD)	63 (24.7)	6 (5.5)	0.704	0.669–10.927	0.163
Number of Smoking friends	None	11 (4.3)	3 (2.7)	1		
	< 10	78 (30.6)	44 (40.0)	0.091	0.011–0.775	0.028
	> 10	43 (16.7)	14 (12.7)	0.431	0.130–1.429	0.169
	Everyone	65 (25.5)	19 (17.2)	0.346	0.083–1.445	0.146
	Not sure	58 (22.7)	30 (27.2)	0.594	0.161–2.190	0.435
Awareness of new price	Yes	239 (93.7)	103 (93.6)	1		
	No	16 (6.3)	7 (6.4)	1.297	0.304–5.538	0.726
Amount after new price	Same amount	149 (58.5)	67 (61.8)	1		
	< 10	95 (37.2)	43 (39.2)	1.485	0.109–20.282	0.767
	> 10	8 (3.2)	0 (0.0)	0.889	0.203–41.050	0.433

**Table 2:** Preferred places for smoking (males)

Places	Category	Current Frequency		*p* value
		Daily (%)	Not daily (%)	
Home	Yes	150 (59.3)	44 (39.3)	< 0.001
	No	103 (40.7)	68 (60.7)	
Work place	Yes	147 (58.1)	32 (28.6)	< 0.001
	No	106 (41.9)	80 (71.4)	
Friend’s house	Yes	144 (56.9)	46 (41.1)	0.005
	No	109 (43.1)	66 (58.9)	
Functions	Yes	134 (53.0)	69 (61.6)	0.125
	No	119 (47.0)	43 (38.4)	
Public places	Yes	40 (15.8)	15 (13.4)	0.552
	No	213 (84.2)	97 (86.6)	
Other	Yes	46 (18.2)	20 (17.9)	0.941
	No	207 (81.8)	92 (82.1)	

### Associations of socioeconomic factors with tobacco smoking according to the awareness of spouses

According to spouses imaginations, frequency of smoking before price increasement, money spend for smoking, proportion spend for smoking from his monthly income, cigarette type tobacco products, low monthly income, monthly income not enough for household activities, friend’s influence for smoking, number of smoking friends, spouse’s employment and education level and husband’s monthly income number of cigarettes smoke per day before and after price increment were significantly associated with tobacco products of their husbands (Table 3).

**Table 3:** Association of socioeconomic factors with tobacco smoking (Female)

Variable	Category	Current Frequency of husbands		OR	95% CI	*p* value
		Daily (%)	Not daily (%)			
Age (Years)	19–39	131 (50.0)	63 (61.2)	1		
	40–59	111 (42.4)	34 (33.0)	1.250	0.280–5.577	0.770
	60–79	20 (7.6)	6 (5.8)	0.786	0.179–3.458	0.750
Educational level	Up to ordinary level	146 (55.7)	43 (41.7)	1		
	Up to advanced level	89 (34.0)	39 (37.9)	0.562	0.215–1.474	0.242
	Diploma / Degree	27 (10.3)	21 (20.3)	0.283	0.060–0.793	0.048
Employment	Government	27 (10.3)	25 (24.3)	1		
	Private	27 (10.3)	13 (12.6)	2.653	1.053–6.687	0.039
	Self-employment	21 (8.0)	7 (6.8)	3.098	1.171–8.195	0.023
	Unemployment	187 (71.4)	58 (56.3)	1.400	0.435–4.503	0.573
Initiation of smoke	Before marriage	177 (67.6)	65 (63.1)	1		
	After marriage	30 (11.5)	14 (13.5)	0.728	0.334–1.587	0.425
	Don’t know	55 (21.0)	23 (22.3)	0.593	0.195–1.808	0.358
Current form of tobacco	Cigarettes	186 (71.0)	92 (89.3)	1		
	Beedi	76 (29.0)	11 (10.7)	0.868	0.277–2.720	0.808
Type/s of premade cigarettes	Gold leaf	109 (58.4)	57 (62.1)	1		
	Bristol	31 (16.4)	17 (18.4)	1.078	0.482–2.413	0.855
	Others	46 (25.2)	18 (19.4)	0.875	0.303–2.528	0.805
Past smoking	Daily	228 (87.0)	35 (34.0)	1		
	Not daily	34 (13.0)	68 (66.0)	0.058	0.029–0.115	< 0.001
Amount of spend per week	< 250	27 (10.3)	10 (9.7)	1		
	250–500	191 (72.9)	65 (63.1)	0.895	0.316–2.532	0.834
	501–1000	44 (16.8)	28 (27.2)	1.217	0.338–4.385	0.764
Husbands monthly income	< 50,000	145 (55.3)	58 (56.3)	1		
	50,001–100,000	59 (22.5)	17 (16.5)	0.320	0.064–1.592	0.164
	> 100,000	58 (22.1)	28 (27.2)	0.469	0.223–0.987	0.046
Proportion spend for smoking from household expenditure	¼	70 (26.7)	20 (19.4)	1		
	½	114 (43.5)	54 (52.4)	0.859	0.400–1.845	0.697
	Other	78 (29.8)	29 (28.2)	1.210	0.468–3.129	0.694
Number of smoking friends	> 10	202 (77.1)	63 (61.2)	1		
	< 10	60 (22.9)	40 (38.8)	0.336	0.171–0.662	0.002
Friend’s influence	Yes	246 (93.9)	90 (87.3)	1		
	No	16 (6.1)	13 (12.7)	0.172	0.057–0.523	0.002
Amount of Cigarettes smoke per day after price increment	< 10	221 (84.3)	95 (92.2)	1		
	> 10	41 (15.7)	8 (7.8)	3.313	1.003–11.059	0.049

Awareness of husband’s preferred place for smoking friend’s house, smoking at home, at work place, functions and public places were significantly associated with tobacco smoking (Table 4).

**Table 4:** Awareness of spouses about preferred places for smoking of their husbands

Places	Category	Current Frequency			*p* value
		Daily	Not daily	Don’t know	
Home	Yes	167 (69.0)	33 (39.8)	15 (37.5)	< 0.001
	No	75 (31.0)	50 (60.2)	25 (62.5)	
Work place	Yes	113 (46.7)	18 (21.7)	10 (25.0)	< 0.001
	No	129 (53.3)	65 (78.3)	30 (75.0)	
Friend house	Yes	117 (48.3)	25 (30.1)	17 (42.5)	0.015
	No	125 (51.7)	58 (69.9)	23 (57.5)	
Functions	Yes	111 (45.9)	45 (54.2)	21 (52.5)	0.023
	No	131 (54.1)	38 (45.8)	19 (47.5)	
Public place	Yes	39 (16.1)	7 (8.4)	11 (27.5)	0.023
	No	203 (83.9)	76 (91.6)	29 (72.5)	
Others	Yes	46 (19.0)	18 (21.7)	9 (22.5)	0.797
	No	196 (81.0)	65 (78.3)	31 (77.5)	

## Discussion

In the present study, the educational level of spouses’ was inversely proportional to the husband’s smoking frequency. It was assumed that higher educational level of the spouses may have more tendencies to distract their husbands from smoking. Mayer et al., (2004) was reported that smoking was significantly lower in men with secondary and higher education compared to those with only primary education [12].

Secondary or higher education may affect to prevent the initiation of smoking or to reduce the frequency of smoking via better awareness about potential health hazards of smoking [13]. Individuals with low level of education have a higher probability to become smokers and higher rates of smoking per day [14]. In the present study, the education is an important predictor of smoking than income. In such circumstances the educational level related differences in smoking might be larger than those related to income or employment. However, Laaksonen et al., 2005 reported that socioeconomic indicators showed a strong association found between socioeconomic indicators and smoking and it was increased gradually from the higher to the lower socioeconomic groups, irrespectively to the indicator they used [15].

Adults in low SES can easily buy tobacco products due to tobacco products are readily available, thus increases the risk of bad health consequences [16, 17]. Low socio-economic condition strongly related to continuation of smoking among adult males, particularly in developing countries [18–20]. Stressful life may strongly affect for the initiation and the higher rates of continuation of smoking. In addition, unemployed adults and adolescents may tend to high continuation rates of smoking due to the effect of low income and high stress condition [21]. Less access to adequate health care and financial difficulties increase their stress levels, making them more susceptible to involve health risks such as smoking [13].

In the present study, the majority of individuals initiated smoking before the age of 18 years old has a high probability to become daily smokers. Similarly studies in Malaysia and China reported that the majority of male smokers imitated smoking by the age of 18 [22, 23]. Similarly a study in Ghana reported that 76.3% of smokers started smoking at age between 16 and 20 years and 80.5% were influenced by friends [24]. Several studies revealed that peer influence is one of the major causes of smoking and often victims started smoking at a very early age in their life [25, 26].

Cigarette is the prominent form of tobacco product in Sri Lanka and the brand of commonest cigarette type was Gold leaf. However, small number of smokers used more than one type of tobacco products such as Cigarette, Beedi, White Beedi, Pipes and Cigar. Beedi is a hand-rolled, leaf- wrapped cigarette, often with sweet flavors. Cigar is a tightly rolled bundle of tobacco wrapped in leaf tobacco. Since cigarettes in Sri Lanka are now close to the most expensive in Asia, more and more adult smokers aged more than 50 years old are turning to smoking Beedis. In the present study, small numbers of male participants use more than one type of premade cigarettes, namely Redrose, Marlboro, Benson, Dunhill, Capton, Sportsman, Three roses and Hedges. Similarly, cigarettes and hand-rolled tobacco were the most commonly consumed tobacco product in Malaysia, Philippines (97.8%) and Thailand (64.9%) male smokers [22, 27, 28]. However, cigarettes were less popular in India (43.1%) and compare to Sri Lanka where hand-rolled tobaccos were commonly used [29].

According to majority of spouses, peer influence has been a major impact on both initiation and maintenance of tobacco smoking habits of their husbands. According to spouses, majority of husband’s used premade cigarettes and the commonest type of cigarette was Gold leaf. Bristol is the second mostly using a type of cigarette. The majority of daily smokers in Sri Lanka were not bounded to any one form or a brand of tobacco and they used more than one form at different times.

In the present study, the average number of cigarettes smoked by the study respondents was 12.3 cigarettes per day. This average number of cigarettes is more than the 11.3 cigarettes per day reported in Philippines [27] and less than 13.5 and 14.3 cigarettes per day reported in China and Vietnam, respectively [30, 31]. Raising the price of cigarette through increasing taxes is a more effective tobacco control policy measure for reducing smoking behavior among young adults and persons of low socioeconomic status [32].

Two thirds of female participants reported that husbands have continued similar rate of smoking daily even after the price increment. Home, at a friend’s house, at parties and at working places were the preferred places for smoking. Home seems like is the safest for our respondents as they might have more freedom. Majority of females mentioned that their husbands preferred to smoke at home. Similarly, in Jordan, one third of husbands from their population smoke inside of their homes [33].

Majority of both participants mentioned only the “home” as preferred place. It reveals that the spouses’ awareness of husband’s habits and behaviors outside of the house is in a low rate. However, in the present study, lower rate of males preferred to smoke in public places. Only 20% of spouses reported that their husbands prefer to smoke in public places. This confirms that they have a certain amount of control of their smoking patterns and behaviors in the public places. This could be a result of the law from NATA Act 2016 “Smoking in public was prohibited in Sri Lanka” or may be the changed attitudes of society.

Majority of the smokers were apparently seen the warning signs of cigarette packages, but those smokers cannot read, have seen the pictorial warnings. Government legislation of warning signs on cigarette packages (80% from the cigarette package) was not very much affected by the reduction of tobacco smoking. Tobacco taxation, passed on to consumers in the form of higher cigarette prices, has been recognized as one of the most effective population-based strategies for decreasing the frequency of tobacco smoking. Tobacco taxes can benefit smokers who quit, reduce the overall consumption of tobacco, and put smoking cessation on their radar of those who continue to smoke. Increased taxes also have a positive impact on non-smokers by reducing their exposure to second-hand smoke. Increased tobacco taxes, passed on to consumers in the form of higher cigarette prices, provide an economic disincentive to those who smoke or may be contemplating smoking [32]. However, due to high price of branded cigarettes might influence the smokers to find some alternatives which might be more dangerous.

Exposure to the anti-smoking media messages using multimedia is another important measure to motivate smokers to quit. Therefore, the programs on health hazards from smoking and control of tobacco products in community level, at workplaces and schools could effect on reducing health disparities.

## Conclusions

In Sri Lanka, the price increment policy has not affected to change tobacco smoking behaviors. Most of male smokers in this study did not reduce the number of cigarettes after the price increase may be because of their addiction to cigarettes. This study strongly supports that increasing the prices of tobacco products via taxations is not a powerful strategy for achieving the reduction in the rate of the smoking behavior Sri Lankan population. Awareness programs on smoking in community level should be commenced targeting all strata of the population and making them aware about the harmful effects and disease conditions of tobacco use. Anti-smoking campaigns must also be initiated in a broad manner, specially targeting the smoking population as well as their family. Media campaigns for anti-smoking campaigns are also very effective.

## Data Availability

All data generated or analyzed during this study are included in the results section in this article. However, identifying/confidential patient data should not be shared.

## Abbreviations

COPD: Chronic obstructive pulmonary diseases
GN: Grama Niladhari officer
LKR: Sri Lankan Rupees
LMIC: Low and middle-income countries
MOH: Medical officers of Health
NATA: National Authority on Tobacco and Alcohol
PHI: Public Health Inspector
PHM: Public Health Midwife
PHNS: Public Health Nursing Sister
USD: United States Dollars
WHO: World health organization

## References

[1] Leone A, Landini L, Leone A (2010). What is tobacco smoke? Sociocultural dimensions of the association with cardiovascular risk. Curr Pharm Des.

[2] Wilson LM, Tang EA, Chander G, Hutton HE, Odelola OA, Elf JL (2012). Impact of tobacco control interventions on smoking initiation, cessation, and prevalence: a systematic review. J Environ Public Health.

[3] Mukherjea A, Morgan PA, Snowden LR, Ling PM, Ivey SL (2012). Social and cultural influences on tobacco-related health disparities among south Asians in the United States. Tob Control.

[4] World Health Organization. WHO report on the global tobacco epidemic: WHO; 2015. URL http://www.who.int/tobacco/global_report/2015/report/en/ (Accessed 1 Nov 2018)

[5] Warren CW, Sinha DN, Lee J, Lea V, Jones NR (2009). Tobacco use, exposure to second hand smoke, and training on cessation counseling among nursing students: cross-country data from the global health professions student survey (GHPSS), 2005–2009. Int J Environ Res Public Health.

[6] Guindon GE, Boisclair D. Past, current and future trends in tobacco use. Washington, DC: HNP discussion paper: World Bank; 2003. https://openknowledge.worldbank.org/handle/10986/13726

[7] Egbe CO, Peterson I, Weitz AM (2016). Knowledge of the negative effects of cigarette smoking on health and well-being among southern Nigerian youth. Int J Soc Sci Humanity.

[8] Warwick H, Alison D. Smoke: the killer in the kitchen; air pollution in developing countries: ITDG Publishing ISBN 1 85339 588 9. 2004. Available at: https://www.humanitarianlibrary.org/sites/default/files/2014/07/itdg%20smoke%20report.pdf.

[9] Umberson D (1987). Family status and health behaviors: social control as a dimension of social integration. J Health Soc Behavior..

[10] Williams K, Umberson D (2004). Marital status, marital transitions, and health: a gendered life course perspective. J Health Soc Behavior.

[11] Umberson D (1992). Gender, marital status and the social control of health behavior. Soc Sci Med.

[12] Mayer O, Simon J, Heidrich J, Cokkinos D, De Bacquer D (2004). Educational level and risk profile of cardiac patients in the EUROASPIRE II substudy. J Epidemiol Community Health.

[13] Crone MR, Reijneveld SA, Willemsen MC, van Leerdam FJM, Spruijt RD, Sing RADH (2003). Prevention of smoking in adolescents with lower education: a school based intervention study. J Epidemiol Community Health.

[14] West P, Sweeting H, Young R (2007). Smoking in Scottish youths: personal income, parental social class and the cost of smoking. Tob Control.

[15] Laaksonen M, Rahkonen O, Karvonen S, Lahelma E (2005). Socioeconomic status and smoking: analysing inequalities with multiple indicators. Eur J Public Health.

[16] Koopmans JR, Slutske WS, Heath AC, Michael C, Neale MC, Boomsma DI (1999). The genetics of smoking initiation and quantity smoked in Dutch adolescent and young adult twins. Behav Genet.

[17] de Vries H (1995). Socio-economic differences in smoking: Dutch adolescents’ beliefs and behaviour. Soc Sci Med.

[18] Wilkinson AV, Vasudevan V, Honn SE, Spitz MR, Chamberlain RM (2009). Socio demographic characteristics, health beliefs, and the accuracy of cancer knowledge. J Cancer Educ.

[19] Kim SR, Kim OK, Yun KE, Khang YH, Cho HJ (2009). Socioeconomic factors associated with initiating and quitting cigarette smoking among Korean men. Korean J Fam Med.

[20] Leinsalu M, Tekkel M, Kunst AE (2007). Social determinants of ever initiating smoking differ from those of quitting: a cross-sectional study in Estonia. Eur J Pub Health.

[21] National Center for Chronic Disease Prevention and Health Promotion (US) Office on Smoking and Health. Preventing tobacco use among youth and young adults: a report of the surgeon general. Atlanta (GA): Centers for Disease Control and Prevention (US); 2012. 22876391

[22] Lim HK, Ghazali SM, Kee CC, Lim KK, Chan YY, Teh HC (2013). Epidemiology of smoking among Malaysian adult males: prevalence and associated factors. BMC Public Health.

[23] Li W, Hsia J, Yang GH (2011). Prevalence of smoking in China in 2010. N Engl J Med.

[24] Yidana A, Boakye-Yiadom A, Osei M (2016). Tobacco use and its socio-cultural dimension among male adults in northern Ghana. Public Health Res.

[25] Conrad KM, Flay BR, Hill D (1992). Why children start smoking cigarettes: predictors of onset. British J Addict.

[26] Bhat M (2014). Tobacco use and awareness patterns among students of an industrial training Institute in Mangalore, South India. Int J Biomed Res.

[27] Global Adult Tobacco Survey Collaborative Group, 2010a. Philippine’s Country Reports. https://www.who.int/tobacco/surveillance/2009_gats_report_philippines.pdf?ua=1. Accessed 5 Feb 2011.

[28] Global Adult Tobacco Survey: Thailand Country. Report http://www.searo.who.int/tobacco/surveillance/Global_Adult_Tobacco_Survey_Thailand_Report_2011.pdf. Accessed 6 Feb 2012.

[29] Global Adult Tobacco Survey (GATS) India Report. 2009, https://www.who.int/tobacco/surveillance/en_tfi_india_gats_fact_sheet.pdf. Accessed 5 Feb 2011), −2010.

[30] Global Adult Tobacco Survey: China Country report 2010. https://www.who.int/tobacco/surveillance/en_tfi_china_gats_factsheet_2010.pdf?ua=1. Accessed 6 Feb 2012.

[31] Global Adult Tobacco Survey Collaborative Group, 2010b. Viet Nam’s Country Reports. https://www.who.int/tobacco/surveillance/en_tfi_gats_vietnam_report.pdf?ua=1. Accessed 6 Feb 2012.

[32] Bader P, Boisclair D, Ferrence R (2011). Effects of tobacco taxation and pricing on smoking behavior in high risk populations: a knowledge synthesis. Int J Environ Res Public Health.

[33] Gharaibeh H, Haddad L, Alzyoud S, El-Shahawy O, Baker NA, Umlauf M (2011). Knowledge, attitudes, and behavior in avoiding second hand smoke exposure among non-smoking employed women with higher education in Jordan. Int J Environ Res Public Health.

